# The Voice of Chinese Health Consumers: A Text Mining Approach to Web-Based Physician Reviews

**DOI:** 10.2196/jmir.4430

**Published:** 2016-05-10

**Authors:** Haijing Hao, Kunpeng Zhang

**Affiliations:** ^1^ Department of Management Science and Information Systems University of Massachusetts Boston Boston, MA United States; ^2^ Department of Decision, Operations & Information Technologies University of Maryland, College Park College Park, MD United States

**Keywords:** online doctor review, physician ratings, text mining, China health consumers

## Abstract

**Background:**

Many Web-based health care platforms allow patients to evaluate physicians by posting open-end textual reviews based on their experiences. These reviews are helpful resources for other patients to choose high-quality doctors, especially in countries like China where no doctor referral systems exist. Analyzing such a large amount of user-generated content to understand the voice of health consumers has attracted much attention from health care providers and health care researchers.

**Objective:**

The aim of this paper is to automatically extract hidden topics from Web-based physician reviews using text-mining techniques to examine what Chinese patients have said about their doctors and whether these topics differ across various specialties. This knowledge will help health care consumers, providers, and researchers better understand this information.

**Methods:**

We conducted two-fold analyses on the data collected from the “Good Doctor Online” platform, the largest online health community in China. First, we explored all reviews from 2006-2014 using descriptive statistics. Second, we applied the well-known topic extraction algorithm Latent Dirichlet Allocation to more than 500,000 textual reviews from over 75,000 Chinese doctors across four major specialty areas to understand what Chinese health consumers said online about their doctor visits.

**Results:**

On the “Good Doctor Online” platform, 112,873 out of 314,624 doctors had been reviewed at least once by April 11, 2014. Among the 772,979 textual reviews, we chose to focus on four major specialty areas that received the most reviews: Internal Medicine, Surgery, Obstetrics/Gynecology and Pediatrics, and Chinese Traditional Medicine. Among the doctors who received reviews from those four medical specialties, two-thirds of them received more than two reviews and in a few extreme cases, some doctors received more than 500 reviews. Across the four major areas, the most popular topics reviewers found were the experience of finding doctors, doctors’ technical skills and bedside manner, general appreciation from patients, and description of various symptoms.

**Conclusions:**

To the best of our knowledge, our work is the first study using an automated text-mining approach to analyze a large amount of unstructured textual data of Web-based physician reviews in China. Based on our analysis, we found that Chinese reviewers mainly concentrate on a few popular topics. This is consistent with the goal of Chinese online health platforms and demonstrates the health care focus in China’s health care system. Our text-mining approach reveals a new research area on how to use big data to help health care providers, health care administrators, and policy makers hear patient voices, target patient concerns, and improve the quality of care in this age of patient-centered care. Also, on the health care consumer side, our text mining technique helps patients make more informed decisions about which specialists to see without reading thousands of reviews, which is simply not feasible. In addition, our comparison analysis of Web-based physician reviews in China and the United States also indicates some cultural differences.

## Introduction

Finding information about health care or health care providers through Web-based platforms has been increasing in recent years. According to the 2013 Health Online Report by the Pew Research Center, about 58% of American adults have used the Internet to seek health-related information at least once in the past year. Nearly half (47%) of adults in the United States have searched for their health providers online, 37% have consulted physician-rating sites, and 7% of people who sought information about their health care providers posted one review online [1]. A new study in the United States also found that 59% of survey respondents said that online doctor ratings are “somewhat important” for them, while 19% said they are “very important” when they search for physicians [2]. Similarly, in the Netherlands, about a third of the Dutch population searches for ratings of health care providers [3]. A study of seven European countries showed that, among the people who use the Internet for health-related purposes, more than 40% considered the information provided by these eHealth services to be important when choosing a new doctor [4]. Besides survey studies on the proportion of the population that has used online ratings of health care providers, researchers have studied how people look at or evaluate those online reviews. Research has shown that review style and number of reviews also has an impact on how patients evaluate those online reviews and on patient attitude toward doctors who received reviews online [5].

Health care researchers have examined the phenomena of online doctor ratings quantitatively. One study showed that about 17% of American physicians have been rated on the Internet. Among them, obstetrician/gynecologists were twice as likely to be rated than other specialists [6]. In Germany, 37% of all German physicians were rated on the jameda website in 2012, and most of the rated medical specialties were orthopedists, dermatologists, and gynecologists [7]. In the United Kingdom, 61% of family practice physicians on the National Health Service Choices website were rated, and 69% of ratings showed that patients would recommend their family doctors. Doctors who practice in a larger facility, with a lower proportion of older patients, lower deprivation, higher population density, and who are not in a solo practice are more likely to be rated. Doctors who serve in smaller size facilities but not in a solo practice, with a higher proportion of white patients, lower population density, and patients who are less deprived are more likely to have a higher level of recommendation [8]. In China, about 37% doctors who registered on the “Good Doctor” platform have been reviewed [9]. However, some medical practitioners or health care researchers argue that online reviews might be skewed because the outspoken angry patients are more likely to rate their doctors online. An empirical study showed that physicians who received lower ratings in surveys are less likely to be rated online, but online doctor ratings are positively correlated with patient opinions from surveys and tend to exaggerate at the higher end of the rating spectrum [10]. In China, the majority of quantitative reviews (star ratings) were positive—88% were positive for the doctors’ treatment effect measure and 91% were positive for the bedside manner measure [9]. In the United States, most online reviews were quite positive, with an average score 3.93 on a scale of 1-5 [6]. In Germany, two thirds of all ratings are in the best category (very good) [7]. In the United Kingdom, the majority (64%) of the online ratings on their National Health Service Choices website are positive [8].

Besides the quantitative ratings associating with reviews, there are also a large number of online textual reviews about health care providers. They can help both health care providers and researchers understand more about patient opinions about care. Unlike the quantitative scores, textual reviews give patients subjective flexibility and freedom to express opinions on their own experiences and concerns. Analysis of online reviews has already been studied in many other domains. For example, mining product reviews has been quite common and successful in the marketing research or management science area, such as using consumer-generated product reviews to analyze people’s online product choices behavior [11], or market structure [12]. However, only a few studies have focused on using text-mining techniques to examine and analyze such largely available textual reviews. One previous US study incorporated latent sentiment analysis into regression analysis and improved state-level health outcome measures [13]. Another study in the United Kingdom applied machine-learning techniques to reviews about hospital service and showed that reviews can be used to predict patient opinions about hospital performance [14]. Based on the authors’ knowledge, only one study has employed an automatic text-mining method to capture hidden topics that health care consumers discussed about their health care providers. In particular, they analyzed online doctor reviews in four specialties in New York City: Family/General Practitioner, Dentist, Obstetrics/Gynecology, and Psychiatrist [15]. However, those studies were restricted by the limited number of available reviews.

In this paper, we intend to apply a well-known text-mining method, Latent Dirichlet Allocation (LDA), to examine what Chinese patients said about doctors or health care services by analyzing a large empirical dataset collected from the largest online health community in China, the Good Doctor platform. To the best of our knowledge, there is no empirical study about what Chinese health consumers say about their health care providers online in spite of the fact that China has over half a billion Internet users—the largest population of Internet users in the world [16]—and is already known to have more than one million online reviews of Chinese doctors [17]. This study explores the following research questions: What do Chinese patients say about their doctors online? Do those topics vary across specialty areas? What can health care providers, health care administrators, or policy makers learn from those million reviews? This is particularly important since China’s health system has been under reform. In addition, are there any differences in reviews of patient care between China and United States?

### Status of Online Doctor Reviews in China

Several online doctor-rating platforms have been created and widely used in China in the past decade. Chinese Medicine Review [18], created in December 2013, focuses on reviewing Chinese traditional medicine doctors. Schedule Web Appointment [19], established in 2010, focuses on online appointment scheduling with doctors across China and is also a platform for reviewing doctors. Among these sites, the “Good Doctor Online” [20] (called “Hao Dai Fu” in Chinese; “Hao” means “good” and “Dai Fu” means “doctor” in Chinese) is the first online doctor review platform in China, initiated in 2006. The Good Doctor is not only the earliest online platform to allow patients to rate and comment on their doctors in many specialty areas, but also the largest one, with more than 300,000 doctors reviewed and one million online reviews [17]. In addition, it provides comprehensive online health-related services such as online appointment scheduling, teleconsultation, patient-doctor forums, and patient clubs for specific doctors.

The Good Doctor was founded with the purpose of helping Chinese health care consumers find “good” doctors for their health-related problems. This is particularly important given that China’s health care system has changed substantially, and China has not built any effective referral system since the 1980s. Most people in China have no primary care providers, and Chinese patients usually self-refer to any providers they can afford or they believe to be good [21]. Before the Internet, Chinese consumers either tried their luck to select a doctor randomly or depended on word-of-mouth recommendations. However, many people do not have friends with the same health problems and do not know which doctors or specialist they should see for their health needs. As a result, Chinese consumers face many difficulties while choosing a “good” doctor. In addition, China is short of doctors, as many countries are. The number of physicians per 1000 residents is 1.8 in 2011 for China, 2.5 in the United States, and 2.8 in the United Kingdom [22]. Therefore, in China, it is extremely difficult to get a walk-in visit with a doctor and even harder to see a good specialist in a popular hospital. Many patients or their family members need to go to the hospital very early in the morning to line up because online scheduling or phone appointments are not widely used in China.

### The Good Doctor Platform

Since being founded in 2006, the Good Doctor platform has been collecting information about Chinese doctors: demographic information, specialty areas, and technical titles, as well as the associated hospital affiliations, such as name, address, and rank level of the hospital. Technical titles are assigned through an evaluation process under a nationally unified ranking system. It has four levels—from junior to senior—from Resident Physician, Attending Physician, Associate Physician, to Chief Physician. On average, every 5 years a doctor can move one level up in this system. Thus, a title primarily indicates a doctor’s work experience and technical skills, which also determines the consultation fee for patients. China’s hospital grades are evaluated and determined by a government agency—the National Health Department at the provincial level—and the evaluation standards are based on the hospital facilities, number of beds, technical equipment, quality of care, the doctors’ skills, etc [23].

Once a doctor’s information is posted on the Good Doctor website, patients can anonymously review the doctors online based on their experiences with those doctors. There are three dimensions on which people can evaluate their doctors on this site: two quantitative measures and one qualitative measure. The two quantitative measures are evaluations of a doctor’s treatment outcomes and bedside manners on a 5-level scale, from “Unsatisfied” to “Very Satisfied.” The qualitative open-ended textual review can be any description or experience associated with the doctor. In order to control for abusive, inappropriate, or fake reviews, those who leave evaluations online are required to provide phone numbers, seen only by the website administrators, so that the site can confirm the veracity of any questionable reviews.

## Methods

### Data

We collected 773,279 public reviews from 112,873 doctors on the platform as of April 11, 2014. In total, there were 314,624 doctors from over 3000 hospitals across China on the site. Thus, about 36% of doctors has been rated or commented on by Chinese patients, which has similar rate to that of German doctors’ online reviews, 37% [7], but higher than that in the United States, where only 17% of doctors have been rated [6].

After data cleaning, such as removing reviews with inaccurate or incomplete information, we had 731,543 reviews with quantitative ratings, 772,979 reviews with qualitative texts, and 731,264 reviews with both quantitative and qualitative measures. The Good Doctor website includes 9 different major medical specialty areas plus one, called “others,” referring to all other less common special areas (see Table 1). We chose the top four specialty areas for analysis in this study: Internal Medicine, Obstetrics (OB)/Gynecology (GYN), Pediatrics, and Chinese Medicine, which received about 23%, 13%, 17%, and 12% of all reviews, respectively. They also have a large number of doctors, with approximately 21%, 19%, 14%, and 11% of doctors, respectively. Table 1 shows that the number of reviews on average that each doctor receives in the specialties of Orthopedics, Oncology, Psychiatry, and Oral Medicine are larger than that in Internal Medicine. But the number of doctors in these areas is much smaller than that in Internal Medicine.

**Table 1:** Number of reviews and doctors by specialty areas.

Specialty areas	Reviews, n	Reviews, %	Doctors, n	Doctors, %	Reviews per doctor, n
Oncology	7372	0.95	1323	1.12	5.6
Chinese Medicine	90,127	11.66	12,073	10.21	7.5
OB/GYN and Pediatrics	128,762	16.66	16,575	14.01	7.8
Infectious Diseases	3205	0.41	486	0.41	6.6
Internal Medicine	102,441	13.25	22,473	19.00	4.6
Orthopedic	3865	0.50	498	0.42	7.8
Others	240,099	31.06	36,281	30.67	6.6
Psychiatry	6429	0.83	1056	0.89	6.1
Oral Medicine	16,346	2.11	2679	2.26	6.1
Surgeon	174,302	22.55	24,846	21.00	7.0
Total	772,948	100	118,290	100	6.5

### Topic Modeling

**Figure 1:**
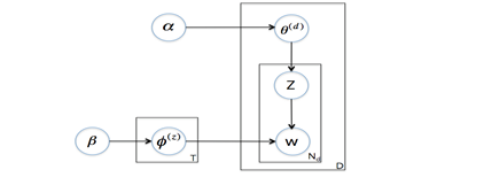
LDA model for topic discovery.

Topic modeling is a sophisticated text-mining technique appropriate for our research task, which is understanding the voice of online Chinese health care consumers by identifying topics on the Good Doctor platform. Topic modeling is a statistical method to uncover abstract topics from a collection of documents [24]. For example, if a document includes flu as a topic, this document is likely to contain related words such as “cold,” “fever,” “cough,” “sneezing”, etc. If a document is about a topic of surgery, then “pain,” “operation,” “surgeon,” “incision,” etc, would co-appear often with high probabilities. Note that the name of the topic is abstracted and summarized by researchers (such as the topics “flu” or “surgery”) based on the most frequently appearing keywords because computer algorithms can find only the pattern of which keywords cluster statistically but cannot summarize what topic those keywords represent. Also, a document usually has a mixture of different topics. Topic modeling can capture those topics in a statistical way by using different algorithms. We used LDA to analyze Chinese consumers’ reviews about their health care providers. LDA has been widely used in various domains, including Web-mining [25], video analysis [26, 27], spam filtering [28], and natural language processing [15, 24, 29, 30]. It is a generative probabilistic model and was first presented for topic discovery by [31], as shown in Figure 1.

*β* is the parameter of the Dirichlet prior on the per-topic word distribution. α is the parameter of the Dirichlet prior on the per-document topic distributions. *θ* ^(d)^is the topic distribution for document *d*(eg, a review), and *z*is the topic assignment for word *w*in a document. *ϕ* ^(z)^is the word distribution for topic *z*. *w*is the word, while *D*is the number of documents. *N*_*d*_is the number of words in a document *d*, and *T*is the number of topics. The LDA model assumes the following generative process for a document *d*=( *w*_*1*_, . . . *w*_*nd*_,) containing *N*_*d*_words from a vocabulary consisting of *V*different terms, *w*_*i*_is the *i* ^*th*^word for all *i*=1, . . . , *N*_*d*_. It consists of the following three steps:

1. The proportions *ϕ*of the word distribution for the topic *z*is determined by *ϕ* ^(z)^~ Dirichlet( *β*).
2. The proportions *θ*of the topic distribution for the document *d*are determined by *θ* ^(d)^~ Dirichlet( *α*).
3. For each of the *N*_*d*_words: (a) Choose a topic *z*~ multinomial( *θ* ^(d)^), and (b) Choose a word *w*_*i*_from a multinomial probability distribution conditioned on the topic *z*: *P*( *w*_*i*_| *z*, *ϕ* ^(z)^)

The number of topics in LDA has to be fixed a priori. In this paper, we split the dataset into two parts: 90% training dataset and 10% testing dataset. The optimal number of topics is determined by the perplexity of the trained model on testing dataset. The key inferential problem that we need to solve in order to use LDA is that of computing a posterior distribution of the hidden variables given in a document: *P(θ, ϕ, z | w, α, β) = P(θ, ϕ, z, w | α, β)*/ *P(w | α, β).*Unfortunately, this distribution is difficult to compute [32]. Although the posterior distribution may not be possible for an exact inference, a wide variety of approximate inference algorithms can be considered for LDA, including varying approximation, Gibbs sampling, and expectation propagation.

The Chinese language is very different from the English language: there are about 3000 basic and commonly used Chinese characters. One single Chinese character usually cannot convey a complete and accurate meaning. At least two or three Chinese characters combined are needed. Therefore, in this study, we need to do some preprocessing before directly applying LDA. We first employ a Chinese character segmentation algorithm implemented by LingPipe [33] to extract meaningful tokens, including removing non-ASCII characters, non-Chinese characters, etc. The extracted tokens may have various lengths from one to a possibly very large number. Each token is considered an atomic entity, meaning that all characters in each token will not be separated for further processing. Then we remove nonsense words, such as stop words in Chinese (eg, of, I, we), and many highly frequent words (eg, doctor, physician, hospital). Finally, we filter out tokens with only one single Chinese character (not meaningful) or those with more than four Chinese characters (likely containing more than one meaning). After this data cleansing, we conduct the following two analyses for each specialty: (1) count the frequency for each unique token, and (2) run the LDA algorithm on reviews for all doctors in each of the four areas to find the top 10 topics, each of which is represented using 10 words with the highest probability within that topic.

## Results

### Descriptive Statistics

**Figure 2:**
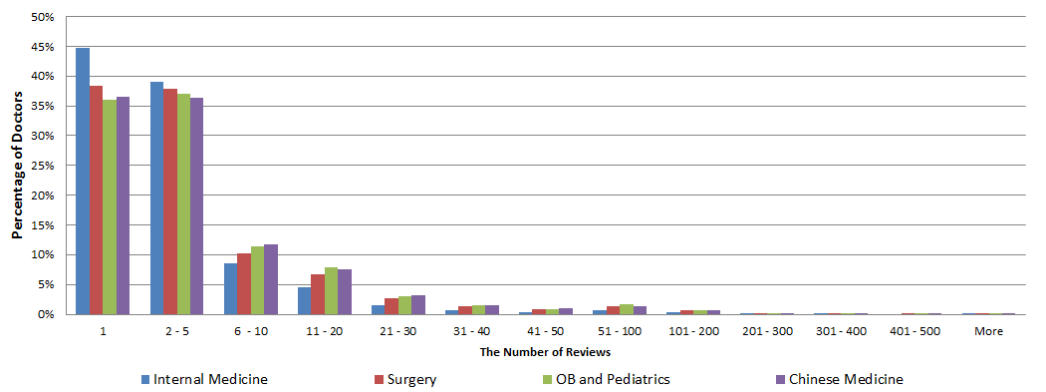
Distribution of the number of reviews a doctor received.

Figure 2 shows the distribution of the percentage of doctors over the number of reviews a doctor received for all four specialty areas. We found that about 36-45% of doctors received one review, 36-39% of doctors received 2- 5 reviews, 8-12% received 6-10 reviews, and 16-27% of doctors received 6 or more reviews. In extreme cases, several doctors received more than 500 reviews. From Figure 2, we also find that the distribution patterns are similar for all four specialty areas.

**Figure 3:**
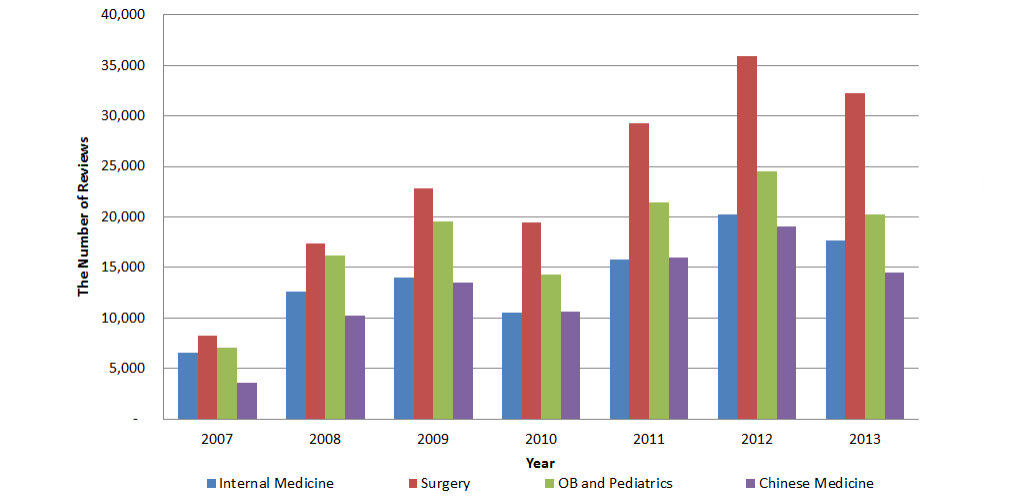
Total number of reviews by specialty over time.

**Figure 4:**
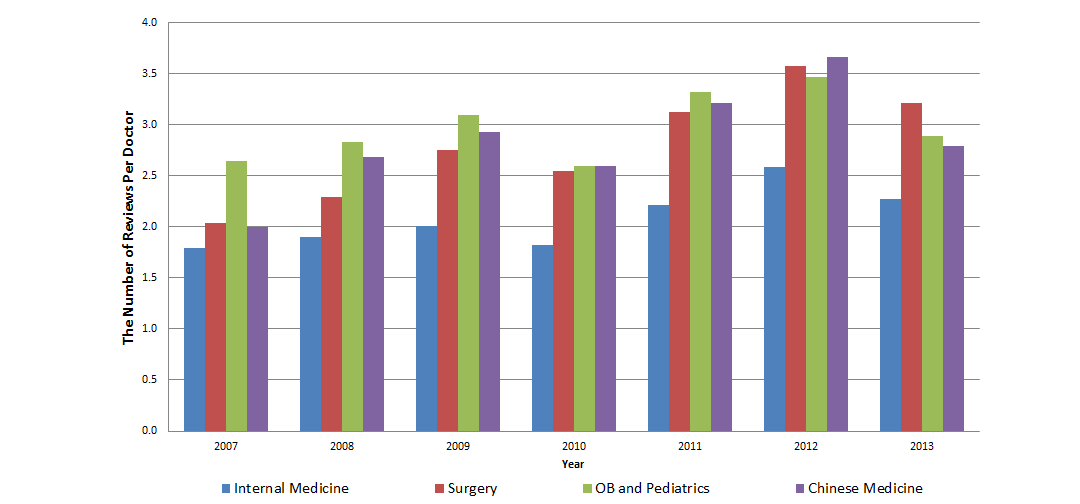
Number of reviews per doctor by specialty over time.

Figure 3 shows the distribution of the review volume from 2007-2013. We did not include reviews for 2006 and 2014 because data are incomplete for the entire calendar year. Figure 3 shows that the number of reviews has been relatively increasing. The trend of the number of reviews per doctor is similar for all four specialty areas over the years as shown in Figure 4.

Table 2 shows the descriptive statistics of review length. If we use regular stoppers to split reviews, such as periods, exclamation marks, or question marks, each review has 3-4 sentences on average for all four specialty areas. Compared to American patients’ average description length, which is about 4 sentences [15], Chinese patients’ reviews seem to be slightly shorter. But, the sentence structure of Chinese is different from English. For example, one Chinese sentence can run very long involving one or more topics separated by commas, which is rare in English. Therefore, to accurately understand the review length, we examine the number of Chinese characters instead. The average number of Chinese characters a review contains is between 85 and 102, which is equivalent to about 40 English words. Based on the authors’ published translated books, the translation rate between Chinese and English is usually 2 to 1, that is, a sentence of 20 Chinese characters can be translated into a sentence of 10 English words. Or if you randomly select a Chinese sentence and put it into Google translation, the translation rate is similar. Table 2 shows that the median length of a review is about 60-70 characters. This indicates more than 50% of the reviews are longer than 30 English words. For some extreme cases, it can be up to over 1700 words for Internal Medicine, OB/GYN, and Pediatrics.

**Table 2:** The descriptive statistics of review length.

	By number of regular stoppers			By number of Chinese characters		
	Average length of reviews	Median length of reviews	Maximum length of reviews	Average length of reviews	Median length of reviews	Maximum length of reviews
Internal medicine	3.3	3	96	85	61	1766
Surgery	3.5	3	81	95	66	1030
OB/GYN and pediatrics	3.6	3	112	101	72	1730
Chinese medicine	3.7	3	77	102	74	1188

### Topic Modeling Result

Based on the LDA algorithm, we identified the top 10 popular topics for each of the four selected specialty areas. Due to space limitations, we present the top 5 popular topics and translate them into English. There are several overlapping or similar topics across specialty areas. The title of each topic is summarized by authors based on the set of keywords returned by the LDA algorithm. For each topic, we show only 10 key words having higher probabilities under that topic. In Tables 3-6, we present the topic summary, the corresponding English translation of the top 10 keywords, and one example review for each topic for the selected four specialty areas, respectively. Based on the assumption of LDA model, each review is a mixture of topics with different probabilities. We select one example review under a topic with the maximum probability.

**Table 3:** Internal medicine.

Topic		Examples
**Topic 1: Treatment effects**		
	Keywords	Now, effect, always , already, better, after, significantly, one time, one year, many years
	Example	I took my mother to see a doctor, and she (mom) had this problem for over 10 years but we never found what caused this problem. Fortunately, we met a student who knew him (doctor) and we visited him for his reputation, diagnosed the problem, and got very good results after.
**Topic 2: Technical skills**		
	Keywords	Medical skill, professional integrity, manner, noble, excellent, super, technique, great, service, experience
	Example	Dr. Liu Shengyun is a good doctor, his consultation process was very thorough, and he has very nice manner, excellent technical skills, and great professional integrity.
**Topic 3: Story of registration**		
	Keywords	Time, out-patient, expert, online, because, saw, consulting, today, registration, visit
	Example	I registered for my sister. It was extreme difficult. I almost died in the crowd because too many people were in the registration window. Luckily I got a registration ticket. My sister doesn’t live in this city. If she comes to register by herself, I guess that she will never get the registration ticket. The medication works well. But she has a swollen face after the medication. At the beginning the doctor said that we should do a follow-up visit in 2 months, so tomorrow morning I will come to fight with the crowd again.
**Topic 4: Bedside manner**		
	Keywords	Patience, manner, serious, responsible, careful, problem, cautious, particularly, every time, warm
	Example	Dr. Liu not only has super great technical skills but is very warm and patient with his patients, as if treating his own friends! He is the best doctor that I ever met.
**Topic 5: Story of finding doctors**		
	Keywords	One, this year, found, introduce, child, Beijing, later, Xiehe hospital, last year, start
	Example	My friend introduced this doctor (to me) and said that this doctor is good for rheumatism. He diagnosed me after reviewing (my) medical record materials and said that only medication may not be ideal, then introduced an intervention doctor, but there were too many patients there, I didn’t have time to ask more questions. Hope next time I will have time to ask.

**Table 4:** Surgery.

Topic		Examples
**Topic 1: Bedside manner**		
	Keywords	Patient, symptom, serious, manner, problem, situation, cautious, very careful, responsible, careful
	Example	Chief Physician Li is very kind, very careful and responsible, and he treats his patients like his own family, very patient, cautious, and he is always very kind, examining carefully, answering questions seriously, and my surgery was very successful. Chief Physician Li is a highly skilled doctor and high professional integrity doctor.
**Topic 2: Appreciate the surgery results**		
	Keywords	Surgery, success, father, mother, removal, check in hospital, surgical
	Example	My father had surgery three days ago, before and after the surgery, Chief Physician Jiao Wenjie with his team gave patients the most comfortable and careful care. As the whole family of the patient, we saw all of that care and wanted to say thanks from the bottom of our heart!
**Topic 3: General appreciation**		
	Keywords	Appreciate, thanks, hello, mom, whole family, hello, hope, health, child, here
	Example	Your amazing hands are a miracle, your kind smiling is as warm as Spring, you are the good luck star for patients, really appreciate you, Dr. Liao Jianna. Best wishes: Good people have a good life.
**Topic 4: Description of symptoms**		
	Keywords	Because, self, know, feel, when, but, always, this, what, many
	Example	I had a bad heart and visited Dr. Li and did some exams, then I knew that I had some problems with aorta valve and atrial fibrillation, then I stayed at hospital, took medication for my heart, until I reached certain conditions, I had surgery.
**Topic 5: Story of finding doctor**		
	Keywords	Time, outpatient, online, found, introduce, expert, saw, friend, shanghai, through
	Example	I have visited all the outpatients that have departments of cerebral surgery in Qingdao, but later a friend introduced Chief Physician Jiao to me, after Chief Physician Jiao’s patient explanation, which helped us laypersons understand the symptoms and the best treatment. Because our budget is tight, Chief Physician Jiao designed a particular operation plan for me to ensure the best surgery outcomes and least cost. This touched us deeply. Chief Physician Jiao is a reliable and respectful good doctor.

**Table 5:** Gynecology/OB and pediatrics.

Topic		Examples
**Topic 1: General appreciation**		
	Keywords	Appreciate, thanks, hello, hope, healthy, health, this, whole family, mom, smooth
	Example	I really appreciate Chief Physician Wang, who cured my child’s decreasing white cells. Nice manner, right medication, cost less. Really appreciate. Wish good health to Chief Physician Wang.
**Topic 2: Story of registration**		
	Keywords	Time, online, expert, because, outpatient, saw, today, visit, therefore, registration
	Example	It is very difficult to get through registration, and only one day out of a week which has 10 registration tickets, and (I) need to line up at 5 o’clock for the registration, and finally get the 18th ticket. One day only has 20 tickets. The doctor is very patient and examines very me carefully, and on average (he can) talk 15 minutes with one patient. Also, the medication is not expensive. It is said that he is an expert of this field, and (I) hope a good doctor like this can have more outpatient time. (I) Trust his technical skills.
**Topic 3: Story of treatment**		
	Keywords	Exam, result, know, what, when, this, what, one time, others
	Example	My child suddenly got laryngotracheal bronchitis, stayed in a hospital for a week, and kept coughing. Every time my child coughs, doctors would say we need to check for asthma. This time when I took my child to get (medical) exam, Dr. Bo was very kind, and also said very definitely that this is not asthma, no need to do other exams, but only a little medicine needed and (ask the child) take it before bed (the child is difficult with taking any medicine). Only two medicines, and each is a half pill, my child was cooperative for taking (this) medicine (in the past, there were several medicines which my child hated), the effect is good, and I really appreciate it! Excellent technical skill and great professional integrity!
**Topic 4: Story of finding doctor**		
	Keywords	Always, pregnant, one time, introduce, found, many, later, period, friend, because
	Example	Because a friend at Provincial Hospital’s introduced me, I visited Dr. Feng. I have an ovarian cyst on the left side and pelvis fluid, so I cannot get pregnant. After one month of medication, the ultrasound shows that both problems are solved. Dr. Feng is a doctor with a parent’s heart. But many times she was too busy to check patients. Later I had 3 months of Gestrinone Capsules. Ultrasound shows both ovarian cyst and pelvis fluid disappeared. But the lower left side of my belly still has a little pain, and when I touched the left side, I could feel something there, it is different from the right side, but the doctor was too busy and only looked at the ultrasound result, did not check. Because of work, I moved to Shanghai, and now over half year, I felt menstrual cramps last month and Shanghai People’s hospital examined and diagnosed it as ovarian cyst. Now after the whole consultation and treatment time, I can say I am exhausted. Doctors at Shanghai said no medication for menstrual cramps. I want to go back to Guangdong for Dr. Feng, her work attitude and technical skills are good, as everyone can see.
**Topic 5: Story of surgery**		
	Keywords	Surgery, uterus, in-patient, year and month, recover, success, fibroids, leave hospital, follow up, found out
	Example	Hello, I am one of your many patients, since I got Uterine fibroids, I have seen many doctors and they all said that I should remove my uterus, I received the recommendation from an acquaintance. I come for the reputation, and Dr. Du of Zhejiang People Hospital is excellent.

**Table 6:** Chinese medicine.

Topic		Examples
**Topic 1: Bedside manner**		
	Keywords	Patience, symptom, manner, serious, cautious, problem, careful, situation, query, every time
	Example	This doctor is a very lovely doctor, my friend accompanied me to see him, once (my friend) saw that this doctor is on duty, and (my friend) got a registration ticket immediately, and said he would be very patient to explain everything.
**Topic 2: Technical skills**		
	Keywords	Manner, technical skill, professional integrity, also, noble, excellent, this, responsible, super, great
	Example	Dr. Ni is a good doctor for his true values. He has a very kind manner to his patients, very nice, very considerate to his patient, excellent technical skills, rich experience! I have been to many hospitals, have seen many doctors, but a doctor like Dr. Ni with such noble professional integrity is hard to find. You can just go and see him, once you see him, you will know what a good doctor means!
**Topic 3: Description of symptoms**		
	Keywords	Many years, myself, serious, because, pain, symptom, mother, cannot, acupuncture, go through
	Example	Appreciate Dr. Sun cured my mom’s arthritis of the shoulder which she had for many years. Also gave me a very targeted treatment. Now, I come to see Dr. Sun again for treatment.
**Topic 4: Story of finding doctors**		
	Keywords	Chinese medicine, online, found, Chinese medicine hospital, year month, at that time, saw, this year, Beijing, found out
	Example	I work in Shanghai and always have stomach problems. Doctors in Shanghai said that is “Reflux Esophagitis” and I have had both western medication and Chinese traditional medication from Level 3 A hospital in Shanghai for almost 5 years, but it didn’t cure it and I had to keep taking medication, which really confused me. I found President Ji of Nantong Chinese Medicine Hospital, so I went to Star Doctor Department at Nantong Chinese Medicine Hospital to see him. President Ji did Gastroscopy for me and found it was “superficial gastritis”. Now I have used his western medication and Chinese medication for about 2 months, I am much better. I am very happy now.
**Topic 5: Concern about child’s health**		
	Keywords	Child, cough, start, son, daughter, cold, baby, later, every time, kid
	Example	Hello, Dr. Hu! Today I will write a belated appreciation letter. Twenty years ago I took my son to see you for his respiratory system asthma, at that time my son was only 2-3 years old, and he got colds frequently, there was a special sound when he coughed, sometimes, and he would get coughing problems 2-3 times in a month. But after seeing you, (after) about 3-4 times in total, (Chinese medicine and acupuncture). Then this problem never came back, sometimes (he) may get cold but no coughing problem any more. Our entire family really appreciates that you cured my son’s problem. Thanks! I always remember your name, if my colleagues or friends’ children have this problem, I will introduce you to them. You are a really high skilled doctor.

**Table 7:** Topics comparison across specialty areas (X means reviews of doctors under a corresponding specialty area largely describe that topic).

	Internal medicine	Surgery	OB-Pediatrics	Chinese Medicine
Treatment effects	X			
Technical skills	X			X
Appreciate the surgery result		X		
Story of treatment			X	
Story of surgery			X	
Bedside manner	X	X		X
Story of registration	X		X	
Story of finding doctors	X	X	X	X
General appreciation		X	X	
Description of symptoms		X		X
Concern about children’s health				X

## Discussion

### Principal Findings

We found some common and distinct topics among the four specialty areas. For example, in Table 7, we can see that the most common topic across four specialty areas is the “story of finding doctors,” which is not a surprise given the following. First, the goal of the Good Doctor platform is to help Chinese patients find good doctors or good specialists for their health problems. Describing how to find good doctors in reviews on this platform should be common. Second, we know that there is no mature primary care systems or professional referral systems in China. This may cause unexpected difficulty for a Chinese patient to figure out which specialist they should see for their medical concerns. Finally, due to the shortage of doctors, 1.8 doctors per 1000 people in China (compared with 2.5 in the United States and 2.8 in the United Kingdom [22]), obtaining a “ticket” for the registration system to see a doctor is always challenging, which results in many complaints. Some of the randomly selected examples in Tables 3-6 also show how difficult it is to see a doctor or how busy a doctor is.

Our findings also show that some topics are quite common and are included across specialty areas, for example, “technical skills” and “bedside manner.” This is not only because they are a focus of patient care, but also that the platform elicits such kinds of reviews. The Good Doctor platform asks reviewers to give rating scores based on these two dimensions before writing text reviews. “General appreciation” and “description of symptoms” are another two common topics across specialty areas. All other topics in the table are found only within one specific specialty. For example, “treatment effects” is seen more in Internal Medicine. Reviews of doctors in Surgery focus on “appreciate the surgery results.” “Concern about children’s health” is reflected more by reviews in the specialty of Chinese Medicine, and this may suggest that Chinese parents prefer to take their children to see Chinese medicine specialists to avoid the potential side effects from western medicine.

We also conducted a comparison between Chinese doctor reviews and American doctor reviews. Topics extracted from both are sometimes different but quite close for similar medical specialties. For example, reviews under Family/General Practitioner in the United States were related more to topics like “manner” and “competence,” while Chinese patients paid more attention to “bedside manner,” “technical skills,” and “treatment effects.” For the specialty area of OB in the United States (OB/GYN and Pediatrics in China), we found that American patients talked more about “manner,” “anecdotal,” “attention,” and “recommendation,” while Chinese patients focused more on various topics, such as “stories on treatment,” “surgery,” “finding doctors,” and “general appreciation.” We also found that many reviewers in the United States recommended doctors explicitly if they were satisfied with their experiences. This may indicate that they consciously realize that other patients may read their posts later. For Chinese patients, they use many polite words to show their appreciation to their doctors directly by addressing the doctor and some also explicitly display their own names and phone numbers, which may indicate that they wish their doctors to recognize them through reviews to receive better treatment next time. Those differences may result from cultural differences. In addition, “attention” is commonly seen in the American doctor reviews. But in this study, we did not specifically have such a topic. It may be included in “bedside manner” and “general appreciation.” Finally, American patients specifically discussed “cost” under the specialty of dentist, and “schedule” for psychiatrist [15]. We did not include these two specialty areas because first, the current Good Doctor platform does not have a separate category called dentist. It is included in the category of oral medicine. Second, the total number of reviews on psychiatrists is too small, with only about 6000 reviews across 1000 doctors.

### Limitations

There are limitations in this study. First, LDA has been used to extract hidden topics [34]. LDA is mainly based on the frequency of co-occurrence of words under similar topics. It might not able to identify some topics that are mentioned by very few reviewers, for example, some emerging topics. Second, our data were collected only from the Good Doctor platform, which might lead to some limitations in data source setting and our methodology. However, the Good Doctor is the largest primary platform of Web-based doctor reviews in China. Also, we want to note that when patients post their reviews on the Good Doctor platform, they are asked to leave their phone number to the webmaster in case any questionable comments need to be verified. This strategy is to prevent any dishonest comments or automatic robotic work. However, some patients might be a little hesitant to make negative comments because of this feature.

### Conclusions

To summarize, Web-based physician review platforms are a good channel for Chinese patients to express their opinions and share their experiences. Topics extracted from those user-generated reviews can provide more understanding of what patients posted. It can also help health care policy makers and health care providers monitor and adjust their policies or resources to better serve their people and improve the quality of health care.

Our study makes several contributions. First, to the best of our knowledge, this is the first text-mining study to understand the voice of Chinese health care consumers by analyzing a large number of Web-based physician reviews. Data were collected from the largest online health care platform in China. Second, we discovered topics from over 500,000 online textual reviews and compared them across specialty areas. Automatic topic analysis provides patients a way to know more about doctors in order to help them make decisions on which specialist they should see. It also helps health care providers or health care policy makers understand patients’ concern or complaints, thus they can adjust their policies or resources to better serve people and improve the quality of health care. Given the fact that the number of online reviews is dramatically increasing, it is simply not practical for a person to read and analyze all the reviews that have been posted. We also compared topics extracted from Chinese doctor reviews and American doctor reviews. Finally, our study empirically demonstrates that Chinese consumers care more about registration, doctors’ bedside manner, and technical skills along with other topics.

There are a few potential studies that could stem from this research. First, we can extend this topic modeling study to Web-based physician reviews for all specialty areas to provide health care providers and researchers with better insight into consumers’ thoughts regarding different medical specialties. Second, we can apply this text-mining technique to similar reviews in other countries to determine whether there are any differences across cultures. Third, it would be interesting to further investigate the relationship between Web-based physician reviews and the quality of care provided by health care professionals.

## References

[1] Kadry B, Chu LF, Kadry B, Gammas D, Macario A (2011). Analysis of 4999 online physician ratings indicates that most patients give physicians a favorable rating. J Med Internet Res.

[2] Hanauer DA, Zheng K, Singer DC, Gebremariam A, Davis MM (2014). Public awareness, perception, and use of online physician rating sites. JAMA.

[3] Van de Belt TH, Engelen LJ, Berben SA, Teerenstra S, Samsom M, Schoonhoven L (2013). Internet and social media for health-related information and communication in health care: preferences of the Dutch general population. J Med Internet Res.

[4] Santana S, Lausen B, Bujnowska-Fedak M, Chronaki C, Kummervold PE, Rasmussen J, Sorensen T (2010). Online communication between doctors and patients in Europe: status and perspectives. J Med Internet Res.

[5] Grabner-Kräuter S, Waiguny MKJ (2015). Insights into the impact of online physician reviews on patients' decision making: randomized experiment. J Med Internet Res.

[6] Gao GG, McCullough JS, Agarwal R, Jha AK (2012). A changing landscape of physician quality reporting: analysis of patients' online ratings of their physicians over a 5-year period. J Med Internet Res.

[7] Emmert M, Meier F (2013). An analysis of online evaluations on a physician rating website: evidence from a German public reporting instrument. J Med Internet Res.

[8] Greaves F, Pape UJ, Lee H, Smith DM, Darzi A, Majeed A, Millett C (2012). Patients' ratings of family physician practices on the internet: usage and associations with conventional measures of quality in the English National Health Service. J Med Internet Res.

[9] Hao H (2015). The development of online doctor reviews in China: an analysis of the largest online doctor review website in China. J Med Internet Res.

[10] Gao G, Greenwood B, Agarwal R, McCullough J (2015). Vocal Minority and Silent Majority: How Do Online Ratings Reflect Population Perceptions of Quality?. MIS Quarterly 2015.

[11] Archak N, Ghose A, Ipeirotis P (2011). Deriving the Pricing Power of Product Features by Mining Consumer Reviews. Management Science.

[12] Netzer O, Feldman R, Goldenberg J, Fresko M (2012). Mine Your Own Business: Market-Structure Surveillance Through Text Mining. Marketing Science.

[13] Wallace B, Paul MJ, Sarkar U, Trikalinos T, Dredze M (2014). A large-scale quantitative analysis of latent factors and sentiment in online doctor reviews. J Am Med Inform Assoc.

[14] Greaves F, Ramirez-Cano D, Millett C, Darzi A, Donaldson L (2013). Use of sentiment analysis for capturing patient experience from free-text comments posted online. J Med Internet Res.

[15] Brody S, Elhadad N (2010). Detecting Salient Aspects in Online Reviews of Health Providers.

[16] The World Bank.

[17] The Economist website.

[18] Chinese Medicine Review.

[19] Schedule Web Appointment.

[20] The Good Doctor.

[21] Eggleston K, Ling Li, Qingyue M, Lindelow M, Wagstaff A (2008). Health service delivery in China: a literature review. Health Econ.

[22] (2011). World Health Organization's Global Health Workforce Statistics.

[23] National Health and Family Planning Commission (1998). Hospital Grade Level Management Evaluation.

[24] Brody S, Elhadad N (2010). An unsupervised aspect-sentiment model for online reviews.

[25] Mei Q, Liu C, Su H, Zhai C (2006). A Probabilistic Approach to Spatiotemporal Theme Pattern Mining on Weblogs.

[26] Niebles J, Wang J, Li F (2006). Unsupervised Learning of Human Action Categories Using Spatialtemporal Words. Proc BMVC.

[27] Wang Y, Sabzmeydani P, Mori G (2007). Unsupervised Activity Perception by Hierarchical Bayesian Model. Proc CVPR.

[28] Biro I, Siklosi D, Szabo J, Benczur A (2009). Linked Latent Dirichlet Allocation in Web Spam Filtering.

[29] Boyd-Graber J, Blei D (2009). Syntactic Topic Models. NIPS.

[30] Blei D, Griffiths T, Steyvers M, Tenenbaum J (2005). Integrating Topics and Syntax. Proc. Neural Information Processing Systems.

[31] Blei D, Ng A, Jordan M (2003). Latent Dirichlet Allocation. Journal of Machine Learning Research.

[32] Dickey J (1983). Multiple hypergeometric functions: Probabilistic interpretations and statistical uses. Journal of the American Statistical Association.

[33] Teahan W, Wen Y, McNab R, Witten I (2000). A compression-based algorithm for Chinese word segmentation. Computational Linguistics.

[34] Tang J, Meng Z, Nguyen X, Mei Q, Zhang M (2014). Understanding the limiting factors of topic modeling via posterior contraction analysis.

